# Preference for Visiting Emergency Department Over Primary Health Care Center Among Population in Saudi Arabia

**DOI:** 10.7759/cureus.20073

**Published:** 2021-12-01

**Authors:** Nasser Almulhim, Fahad Almulhim, Ali Al Gharash, Zahra Alghannam, Rami S Al-ghamdi, Mohammed H Alghamdi, Ali H Alghareeb, Abdulaziz Y Alabdulrahman

**Affiliations:** 1 Emergency Medicine, King Fahad Hospital, Al-Hofuf, SAU; 2 Medicine, King Faisal University, Al-Hofuf, SAU

**Keywords:** saudi arabia, quick medical care, primary health care, emergency room, emergency departments

## Abstract

Introduction

Demand for urgent care is increasing, and the pressure on emergency departments (EDs) is of significant concern. Demand growth is to some extent due to the over-utilization of EDs by patients who seek care for non-urgent problems. It has been estimated that up to one-third of all ED visits are non-emergent. In EDs, patients with non-urgent problems are blamed for increased demand, even though most of the patients’ reasons for attending EDs are not well studied. The aim of this study is primarily to determine the factors that influence the decision of patients to visit EDs instead of their primary care physician for non-urgent problems. Secondary aim of this study is to assess the level of ED knowledge among the participants.

Methods

This is a descriptive, cross-sectional study following a convenient sampling technique conducted through an online questionnaire distributed to the population in Saudi Arabia (SA). The data, which includes sociodemographic data, ER knowledge, the correct definition of ED, cases that ED deals with, and reasons for visiting an ED over a primary health care center (PHC), were collected during August and September 2021.

Results

Of the 915 respondents, the most common age group was 25 years old or less (34.4%) and the majority were females (68.3%). It was observed that slightly more respondents preferred to visit a PHC (50.4%) while 49.6% preferred to visit EDs when having a medical condition. The proportion of respondents who would visit a PHC was 90.9%, and 36.6% indicated having good experiences there. The overall mean knowledge score was 4.63 (standard deviation [SD] 1.51) out of 6 points, with low, moderate, and high knowledge classified among 12.9%, 22.4%, and 64.7% of respondents, respectively. The five most reported reasons for choosing the ER as the preferred clinic over a PHC were: (1) ED provided quick medical care, (2) easy access to emergency care, (3) unavailability of appointments at a PHC center on the same day, (4) lack of full investigation at the PHC center, (5) lack of primary care providers at the PHC. The least reported reason was advice from another person to visit the emergency department. It was found that a significantly low knowledge was demonstrated by the over 45-years age group (p <0.001) and those who were unemployed (p = 0.018).

Conclusion

This study showed that 49.6% would prefer to visit the ED. It demonstrated the reasons for choosing the ED over a PHC, with the most reported reason being that the ED provides quick medical service, while the least reported reason was advice from another person to visit the ED. For the correct utilization of EDs, it is recommended to increase the knowledge and awareness level of the general population through public campaigns and awareness videos on social media applications.

## Introduction

Demand for urgent care is increasing, and the pressure on emergency departments is of significant concern [1]. Overcrowding in emergency departments has been described as an “international symptom of health-care system failure.” Demand growth is to some extent due to the over-utilization of EDs by patients who seek care for non-urgent problems [2]. It has been estimated that up to one-third of all emergency department (ED) visits are non-emergent [3]. In EDs, patients with non-urgent problems are blamed for increased demand, even though most patients’ reasons for attending EDs are unknown [4]. Many people come directly to EDs to seek fast medical care either for themselves or their children. However, the presence of both non-urgent walk-in patients and acute emergencies can make it more difficult to provide genuine emergencies with rapid treatment, leading to deterioration in the quality of emergency services and higher overall costs [5]. Complications of overcrowding in EDs include increases in morbidity and mortality rates; insufficient time for investigation, treatment, and pain management; difficulties maintaining patient privacy; ambulance diversion and ramping; increased length of stay; decreased staff productivity and increased burnout; increased violence; and miscommunication. These factors may eventually lead to decreased staff and patient satisfaction [4]. This kind of study has not been done before in Saudi Arabia. We should understand the public concerning their characteristics and reasons for presenting at EDs, even for non-urgent problems.

## Materials and methods

This descriptive, cross-sectional study aimed to determine the factors influencing the decision of patients to visit an ED for non-urgent problems instead of a primary health care center (PHC). Ethical approval was granted from the Institutional Review Board committee of King Fahad Hospital in Al-Hofuf. The study involved the digital distribution of questionnaires between August and September 2021 to residents of the Kingdom of Saudi Arabia.

The inclusion criteria for this study were all Saudi and non-Saudi populations living in Saudi Arabia, with no exclusion criteria.

The data for this study were collected by using a standard questionnaire from previously published similar studies with some modifications reviewed by emergency physicians. Then, a pilot study was conducted among a small sample size to ensure the validation and reliability of the questionnaires. A convenience sampling technique was followed in this study. The questionnaire had two sections: the first for sociodemographic data that included questions about age, gender, marital status, occupation, nationality, residency, and level of education, and the second to gauge ED knowledge, the correct definition of an ED, cases that EDs deal with, and reasons for visiting an ED over a PHC for non-urgent treatment.

The sample size was determined by Raosoft software (Raosoft Inc, Seattle, USA), with a margin of error of 5%, confidence level 95%, population size about 10,00,000, and response distribution of 50% for calculation of sample size. The sample size included 400 participants.

The data were analyzed using Statistical Packages for Social Sciences (SPSS) version 26 (IBM Corp, Armonk, NY). Categorical variables were presented as numbers and percentages, and continuous variables as mean and standard deviation. Knowledge about EDs was assessed via the questionnaire. Question one had a single item, Question two was a multiple-response answer with five correct answers, and the total items were six. The total knowledge score was obtained by adding the six items, and a score range from 0-6 was generated, indicating that the higher the score, the higher the knowledge about EDs. Participants were then classified as low knowledge (score 0-2), moderate knowledge (score 3-4), and high knowledge (score 5-6). Participants’ preference of clinic visitation (ED vs. PHC) and knowledge about ED were compared with their sociodemographic characteristics by using the Chi-square test. A p-value cut-off point of 0.05 at 95% CI was used to determine statistical significance.

## Results

There were 915 respondents. Table 1 describes the sociodemographic characteristics of the sample population. The most common age group was 25-years-old or less (34.4%), the majority were females (68.3%), and 37.8% were working.

**Table 1:** Participants’ sociodemographic characteristics according to the preferred clinic § P-value was calculated using the Chi-square test ** Significant at p <0.05 level ED: Emergency department, PHC: Primary health care center

Variables	Overall N (%) ^(n=915)^	Preferred clinic		P-value ^§^
		ED N (%) ^(n=454)^	PHC N (%) ^(n=461)^	
Age group				
≤25 years	315 (34.4%)	147 (32.4%)	168 (36.4%)	0.086
26–35 years	137 (15.0%)	60 (13.2%)	77 (16.7%)	
36–45 years	209 (22.8%)	117 (25.8%)	92 (20.0%)	
>45 years	254 (27.8%)	130 (28.6%)	124 (26.9%)	
Gender				
Male	290 (31.7%)	128 (28.2%)	162 (35.1%)	0.024 **
Female	625 (68.3%)	326 (71.8%)	299 (64.9%)	
Occupation				
Student	259 (28.3%)	114 (25.1%)	145 (31.5%)	0.123
Unemployed	110 (12.0%)	53 (11.7%)	57 (12.4%)	
Employed	346 (37.8%)	186 (41.0%)	160 (34.7%)	
Housewife	200 (21.9%)	101 (22.2%)	99 (21.5%)	
Educational level				
Diploma or below	351 (38.4%)	173 (38.1%)	178 (38.6%)	0.875
Bachelor’s degree or higher	564 (61.6%)	281 (61.9%)	283 (61.4%)	
Marital status				
Unmarried	350 (38.3%)	164 (36.1%)	186 (40.3%)	0.189
Married	565 (61.7%)	290 (63.9%)	275 (59.7%)	
Nationality				
Saudi	904 (98.8%)	449 (98.9%)	455 (98.7%)	0.781
Non-Saudi	11 (01.2%)	05 (01.1%)	06 (01.3%)	
Living in Al-Ahsa				
Yes	696 (76.1%)	346 (76.2%)	350 (75.9%)	0.918
No	219 (23.9%)	108 (23.8%)	111 (24.1%)	
Residence location				
Village	264 (28.9%)	131 (28.9%)	133 (28.9%)	0.999
City	651 (71.1%)	323 (71.1%)	328 (71.1%)	

Table 2 shows the characteristics of participants who visited an ED or a PHC. It can be observed that slightly more respondents would visit a PHC (50.4%), while 49.6% would visit the ED. About 41.4% of the respondents would usually visit the ED in the morning (41.4%) or the afternoon (25.8%). The proportion of respondents who visited a PHC was 90.9%, and 36.6% indicated good experiences when doing so.

**Table 2:** Participants’ sociodemographic characteristics according to the preferred clinic § P-value was calculated using the Chi-square test ** Significant at p <0.05 level

Variables	N (%)
When you have a medical condition, do you prefer to visit	
Emergency department	454 (49.6%)
Primary health-care center	461 (50.4%)
What time do you visit the emergency department most often?	
In the morning	379 (41.4%)
In the afternoon	236 (25.8%)
Don’t prefer visiting the emergency department	300 (32.8%)
Have you ever visited a primary health care center?	
Yes	832 (90.9%)
No	83 (09.1%)
Rate your primary health care center experiences ^(n=832) †^	
Excellent	164 (19.7%)
Good	300 (36.1%)
Acceptable	119 (14.3%)
Need improvement	249 (29.9%)

The assessment of knowledge about ED is shown in Table 3. Following the results, it was revealed that 81.2% of the respondents were aware that the correct description of ED was “it is part of a hospital that provides 24-hour emergency care to patients who need urgent medical attention.” Furthermore, there were proposed emergency cases to be classified as emergencies which revealed good knowledge. The most common cases listed were “A road traffic accident associated with continuous bleeding from right leg” (89.8%), followed by “Chest pain associated with sweating and shortness of breath” (83.2%), “Taking many Paracetamol tablets when attempting suicide” (76.1%), “Abdominal pain associated with bloody diarrhea and vomiting” (71.6%), and “Weakness in the right/left lower limb with slurred speech” (61.3%). Based on the above statements, the overall mean knowledge score was 4.63 (SD 1.51) out of 6 points with low, moderate, and high knowledge classified among 12.9%, 22.4%, and 64.7% of respondents, respectively. 

**Table 3:** Assessment of knowledge about ED (n=915) ‡ Variable with multiple response answers * Indicates correct answer ED: Emergency department, SD: Standard deviation

Variables	N (%)
Which of the following statements describes the emergency department?	
‎It is part of a hospital that provides 24-hour emergency care to patients who need urgent medical attention *	743 (81.2%)
Is the first place to visit when people develop a medical condition	113 (12.3%)
I don’t know	300 (32.8%)
Knowledge about the cases classified as emergency ^‡^	
A road traffic accident associated with continuous bleeding from the right leg *	822 (89.8%)
Chest pain associated with sweating and shortness of breath *	761 (83.2%)
Taking many paracetamol tablets to attempt suicide *	696 (76.1%)
Abdominal pain associated with bloody diarrhea and vomiting *	655 (71.6%)
Weakness in the right/left lower limb with slurred speech *	561 (61.3%)
Testing COVID-19 positive without shortness of breath	163 (17.8%)
Sore throat with fever	119 (13.0%)
Diarrhea not associated with blood, abdominal pain, and fever	76 (08.3%)
Itchy nose and sneezing	53 (05.8%)
Knowledge total score (mean ± SD)	4.63 ± 1.51
Low	118 (12.9%)
Moderate	205 (22.4%)
High	592 (64.7%)

**Figure 1:**
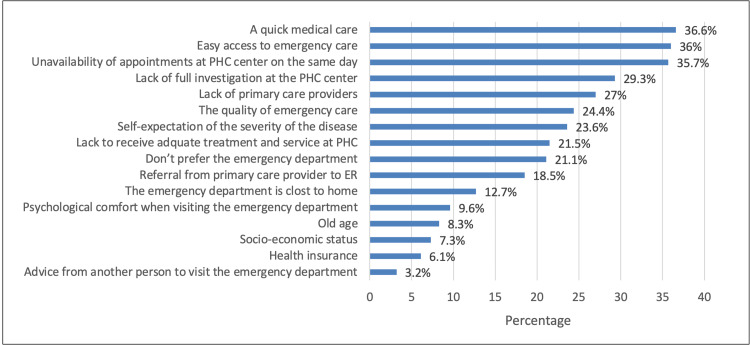
Reason for choosing ED as a preferred clinic over PHC ED: Emergency department, ER: Emergency room

Figure 1 depicts the reasons for choosing ED as a preferred clinic over a PHC. It can be observed that the top five most common reasons were that ER provides “Quick medical care” (36.6%), followed by “Easy access to emergency care” (36%), “Unavailability of appointments at PHC center on the same day” (35.7%), “Lack of full investigation at the PHC center” (29.3%), and “Lack of primary care providers at the PHC” (27%). “Advice from another person to visit the emergency department” was the least common reason (3.2%).

We used the Chi-square test in Table 4 to determine the relationship between the level of knowledge about ER and the sociodemographic characteristics of participants. Based on the results, it was found that significantly low knowledge was demonstrated by the age group above 45 years (p <0.001) and those who were not working (p = 0.018), while high knowledge was more common among those with a bachelor’s degree or higher (p <0.001). On the other hand, the level of knowledge among gender, marital status, living in Al-Ahsa, and residence location variables was not significantly different across the group (p >0.05). 

**Table 4:** Relationship between the level of knowledge about ED and the sociodemographic characteristics of participants § P-value was calculated using the Chi-square test ** Significant at p <0.05 level ED: Emergency department

Variables	Level of knowledge			P-value ^§^
	Low N (%) ^(n=118)^	Moderate N (%) ^(n=205)^	High N (%) ^(n=592)^	
Age group				
≤25 years	26 (22.0%)	66 (32.2%)	223 (37.7%)	<0.001 **
26–35 years	15 (12.7%)	35 (17.1%)	87 (14.7%)	
36–45 years	25 (21.2%)	35 (17.1%)	149 (25.2%)	
>45 years	52 (44.1%)	69 (33.7%)	133 (22.5%)	
Gender				
Male	39 (33.1%)	66 (32.2%)	185 (31.3%)	0.915
Female	79 (66.9%)	139 (67.8%)	407 (68.8%)	
Occupation				
Student	21 (17.8%)	58 (28.3%)	180 (30.4%)	0.018 **
Unemployed	11 (09.3%)	23 (11.2%)	76 (12.8%)	
Employed	47 (39.8%)	78 (38.0%)	221 (37.3%)	
Housewife	39 (33.1%)	46 (22.4%)	115 (19.4%)	
Education level				
Diploma or below	63 (53.4%)	87 (42.4%)	201 (34.0%)	<0.001 **
Bachelor’s degree or higher	55 (46.6%)	118 (57.6%)	391 (66.0%)	
Marital status				
Unmarried	37 (31.4%)	75 (36.6%)	238 (40.2%)	0.168
Married	81 (68.6%)	130 (63.4%)	354 (59.8%)	
Living in Al Ahsa				
Yes	94 (79.7%)	154 (75.1%)	448 (75.7%)	0.610
No	24 (20.3%)	51 (24.9%)	144 (24.3%)	
Residence location				
Village	27 (22.9%)	68 (33.2%)	169 ( 28.5%)	0.140
City	91 (77.1%)	137 (66.8%)	423 (71.5%)	

## Discussion

One study showed that many patients visit the ED as they consider it to be faster and more convenient [3]. However, in this study, it showed that slightly more respondents prefer to visit the PHC (50.4%) while (49.6%) visit the ED. In Kentucky, a survey showed that patients, in fact, would prefer to see their primary care physicians, but nearly half of them would “seek care elsewhere” if they could not be seen the same day [3], which is observed in this study as one of the most reported reasons for visiting the ED. Of the people who participated in the questionnaire, 832 (90.9%) had visited a PHC and their positive experiences ranged from excellent (164 persons, 19.7%) to good (300, 36.1%) or acceptable (119, 14.3%). A further 249 persons (29.9%) said that their experiences needed improvement.

Several studies have recognized that the perspectives of patients toward using EDs are different [6,7]. In a 2012 study, 262 patients were grouped into the non-emergent category (n = 129) and emergent category (n = 131), according to their perception of the severity of their complaints [3]. Our study assessed knowledge about the ED in the study area (Table 3). Even though there is a lack of a clear, universal definition among patients and health care providers as to just what the term “non-emergent” exactly means, our study revealed that 81.2% of respondents were aware that the correct description of the ED was “It is part of a hospital that provides 24-hour emergency care to patients who need urgent medical attention.” Furthermore, most common ED cases were used to additionally assess the respondents’ knowledge of the ED, and they showed good knowledge about the most common cases that were classified as an emergency, which were: “A road traffic accident associated with continuous bleeding from the right leg” (89.8%), followed by “Chest pain associated with sweating and shortness of breath” (83.2%), “Taking many paracetamol tablets to attempt suicide” (76.1%), “Abdominal pain associated with bloody diarrhea and vomiting” (71.6%), and “Weakness in the right/left lower limb with slurred speech” (61.3%). Even though our study had some limitations, the overall mean knowledge score in the studied population was 4.63 (SD 1.51) out of 6 points, with low, moderate, and high knowledge classified among 12.9%, 22.4%, and 64.7% of respondents, respectively.

Multiple studies have shown various reasons why people prefer to visit EDs over PHCs. However, in our study, 21.1% of 915 participants did not prefer the ED. The remaining 78.9% were allowed to choose more than one reason. Many patients preferred the ED because it provides fast medical care (36.6%), which is consistent with other research that has shown many patients prefer the ED because it is faster and more convenient [8,9]. Also, 36% of ED visits were due to the unavailability of appointments at a PHC for seeking medical care on the same day. Some investigations have shown that the most influential factors include better accessibility and self-perceived severity of complaints, which in our study represented 36% for easy access to emergency care and 23.6% for self-expectations of disease severity [10]. Referrals are another reason for visiting the ED. In our study, a referral from a PHC to the ED represented 18.5% of cases, while another study showed that 81 patients had been referred to the ED out of 200 patients, which is about 40% [11]. In addition, in another study conducted for pediatric ED, of 251 respondents 45.4% showed that they contacted a primary health physician and 77.6% were referred to the ED [11]. More factors, such as “Lack of full investigations at PHC centers” (29.3%), “Lack of primary care providers” (27%), “Quality of emergency care” (24.4%), and “Lack of adequate treatment and service at a PHC” (21.5%) had a significant impact on preference for the ED. Other, less effective factors were “ED is close to home” (12.7%), “Psychological comfort when visiting ED” (9.6%), “Old age” (8.3%), “Socioeconomic status” (7.3%), “Health insurance” (6.1%), and “Advice from others to visit the ED” (3.2%).

The results imply that the respondents with a high level of knowledge mainly comprised those with a bachelor’s degree or higher (p <0.001) and unsurprisingly demonstrated that those having a diploma or below had a low level of knowledge. In contrast, it was found that significantly low knowledge was demonstrated by those over 45 years of age (p <0.001). This means that the younger population has a higher knowledge about the ED than those who are older than 45. Similarly, those who were not working were found to have significantly low knowledge compared with those who have jobs (p = 0.018). Yet there was no significant relationship between the level of knowledge and gender, marital status, living in Al-Ahsa, and residence location (p >0.05).

## Conclusions

More than half of the studied population were found to be knowledgeable about how to describe the ED and could identify the proposed emergency cases. Of the respondents, 915 (50.4%) preferred to visit a PHC, while 49.6% preferred the ED. This study has demonstrated mainly the reasons for choosing the ED over PHC. The most significant reason reported by the study participants was that it provides fast medical care. This is followed by easy access to emergency care, unavailability of appointments at a PHC on the same day, and lack of full investigation at the PHC. On the other hand, 21.1% of the study participants reported that they did not prefer the ED. Even though the studied population demonstrated acceptable knowledge regarding the ED, it is recommended to increase the knowledge and awareness level of the general population for correct utilization of EDs, through public campaigns and awareness videos on social media applications.
